# Socio-ecological analysis of nurses’ roles and challenges in choice on termination of pregnancy services in South Africa: A scoping review

**DOI:** 10.4102/jphia.v17i1.1600

**Published:** 2026-03-23

**Authors:** Vutlhari A. Ndlovu, Tshepo A. Ntho, Masenyani O. Mbombi, Mxolisi W. Ngwenya

**Affiliations:** 1 Department of Nursing, Faculty of Health Science, University of Limpopo, Polokwane, South Africa

**Keywords:** socio-ecological framework, current issues, choice on termination of pregnancy, nurses, barriers, challenges, South Africa, Scoping Review

## Abstract

**Background:**

Choice on termination of pregnancy remains a sensitive and complex aspect of reproductive healthcare, particularly in settings where cultural, religious, legal and institutional factors intersect.

**Aim:**

To identify nurses’ roles and challenges in providing choice on termination of pregnancy services in South Africa using a socio-ecological framework.

**Setting:**

The review was conducted using findings from studies on nurses regarding the provision of choice on termination of pregnancy services in South Africa.

**Method:**

A scoping review was conducted following the Arksey and O’Malley framework and aligned with the Systematic Reviews and Meta-Analyses extension for Scoping Reviews guidelines. Electronic databases include ScienceDirect, EBSCOhost, PsycINFO, PubMed and regional databases like African Index Medicus. Literature published between January 1996 and December 2024 was searched; only English-language studies were included.

**Results:**

Twenty-one studies, published between 1998 and 2023, were included. The results show diverse and in-depth insights into the multifaceted challenges of nurses involved in choice on termination of pregnancy services, highlighting emotional and moral challenges, interpersonal dynamics, organisational constraints, sociocultural influences and legal gaps.

**Conclusion:**

This review reveals that nurses’ roles in providing choice on termination of pregnancy services are shaped by multiple levels of influence, with moral beliefs, stigma and systemic barriers hindering the delivery of quality care.

**Contribution:**

This review enhances understanding of the multi-level barriers nurses face in providing choice on termination of pregnancy services in South Africa and underscores the need for targeted interventions to improve access to sexual and reproductive health services.

## Introduction

Choice on termination of pregnancy (CTOP) is one of the least accessible services of sexual and reproductive health. This limited accessibility is often attributed to nursing-related factors such as negative attitudes, inadequate training and the influence of individual religious or cultural beliefs.^1,2^ Termination of pregnancy (TOP) refers to the deliberate separation and expulsion of the contents of the uterus of a pregnant woman through either medical or surgical means.^3^ Medical TOP uses pharmacological means to induce uterine contractions and expel the uterine contents, whereas surgical TOP involves transcervical procedures, such as vacuum aspiration or dilatation and evacuation.^4^ Both methods are safe and effective and may be provided when continuation of the pregnancy would substantially affect the woman’s social or economic circumstances, pose a risk to her physical or mental health or for other reasons considered clinically or legally appropriate. Notably, over 260 000 nurses are registered with the South African Nursing Council (SANC), making them the most accessible healthcare providers in the country.^5^ Despite this nursing manpower, access to CTOP services remains constrained. Equally important, these constraints on CTOP access are further perpetuated by existing legal frameworks.

According to the National Clinical Guideline for Implementation of the *Choice on Termination of Pregnancy (CTOP) Act*, only registered medical practitioners or trained nurses and midwives are permitted to perform terminations.^4^ In line with this guideline, nurses are allowed to terminate pregnancies up to 12 weeks and 6 days at a woman’s request, without requiring a stated reason.^4^ This restriction is based on the understanding that early TOP procedures carry fewer health risks, are less complex to perform and are more cost-effective for health facilities. Furthermore, nurses involved in CTOP services play a vital role in providing pre- and post-counselling and educating clients on contraceptive methods to prevent future unintended pregnancies.^6,7^ While the South African context presents unique legal and professional dynamics, the accessibility and implementation of CTOP services remain a global public health and social challenge. In Ireland, the main challenges related to CTOP have been identified as a lack of education and training, a shortage of staff and a lack of resources.^8^ Similarly, in Thailand, it has been found that nurses lack knowledge and do not have favourable attitudes towards CTOP.^9^ Evidently, religion, cultural beliefs and attitudes of nurses play a crucial role in the accessibility of TOP services.^10^

Sub-Saharan Africa has made great progress in addressing unsafe CTOP since 2000 through legalisations, regulations and policies.^11^ However, that does not equate to the progress of accessibility of the CTOP service. Evidently, TOP services in countries like Zimbabwe and Kenya remain an option only under exceptional circumstances.^12,13^ Furthermore, countries like Nigeria report a rate of more than 50% of unsafe TOP to date, with most of the women coming from disadvantaged backgrounds.^14^ In Ethiopia, recognised barriers to the access of safe TOP care include costs of services, lack of privacy and fear of judgement from providers. Women who had had negative experiences with providers were more likely to seek unsafe CTOP the second time around.^15^ Equally important, restrictive religious beliefs and deeply rooted social norms across many countries further limit women’s access to safe CTOP services.^16^ From a socio-ecological perspective, social norms at interpersonal and community levels serve as significant barriers to women’s access to safe CTOP services by perpetuating stigma and limiting supportive social environments.

The *CTOP Act* 92 of 1996 replaced the restrictive *Sterilization Act* of 1975, providing safe, early and legal access to termination services. Under the *CTOP Act*, a pregnancy may be terminated if it poses a risk to the woman’s physical or mental health, results from rape or incest or would significantly impact her social or economic circumstances. Despite this legislative advancement and broader efforts to improve women’s sexual and reproductive health, unsafe and informal terminations continue to present significant public health and social challenges. It is estimated that annually, between 52% and 58% of terminations of pregnancy are performed outside the legal framework and are therefore unsafe.^17^ Equally important, fewer than 7% of public health facilities in South Africa are reported to provide CTOP services.^18^ The limited availability of CTOP services in public facilities increases the likelihood that women may resort to unsafe ‘backstreet’ terminations, which pose serious health risks and can lead to severe complications or even death.^18^ This persistent gap between policy and practice underscores the urgent need to interrogate the structural, institutional and professional barriers that hinder the full realisation of reproductive rights.

Nurses are often the first and, sometimes, the only point of contact for women seeking reproductive health services. The barriers mentioned directly undermine access to safe CTOP care, perpetuating the cycle of unsafe TOP despite the enabling legal framework. Examination of the literature affirms that nurses encounter challenges when they want to improve their knowledge and skills through training.^6^ Furthermore, there is a lack of integration of CTOP in current nursing curricula and education, and this delineates a gap in the provision of sexual reproductive health services in South Africa. Choice on termination of pregnancy is a crucial service that requires urgent attention to achieve Sustainable Development Goal (SDG) 3. This review aims to systematically explore and synthesise the literature on nurses’ roles, experiences and challenges in providing CTOP services in South Africa. Using the Socio-Ecological Framework, the review seeks to identify and map factors that influence the delivery and accessibility of safe CTOP care, at individual, interpersonal, organisational, community and policy levels. Equally, this review highlights key areas for intervention and policy improvement to better support nurses, reduce stigma and enhance access to safe CTOP care, ultimately contributing to improved reproductive health outcomes and informing future research, practice and policy development.

## Methods

### Design

A scoping review method was conducted to synthesise the availability and type of evidence available regarding the roles and challenges faced by nurses in providing CTOP services in South Africa. This scoping review was guided by the methodological framework of Arksey and O’Malley.^19^ The framework has six steps, of which only five steps were used in this study in identifying, selecting and extracting data for the review. The stages are as follows: (1) identifying the research question; (2) identifying relevant studies; (3) selecting the studies; (4) charting the data and (5) collating, summarising and reporting the results. In addition, the current review report followed the Preferred Reporting Items for Systematic Reviews and Meta-Analysis Extension for Scoping Review (PRISMA-ScR) guideline has been adhered to in this study.^20^

#### Stage 1: Identifying the research question

The scoping review served as the foundation for this study, aiming to identify, critique, evaluate, synthesise and interpret the available evidence on the roles and challenges faced by nurses in providing CTOP services in South Africa. Additionally, it examined existing literature to identify gaps in the provision of CTOP healthcare services. The study was guided by the following research question:

- *What are the roles and challenges faced by nurses in providing CTOP services in South Africa, as understood through the Socio-Ecological Framework?*

#### Stage 2: Identifying relevant studies

This review examined studies conducted over a 28-year period, with the search parameters set from 01 January 1996 to 31 December 2024. This timeframe is particularly significant as it aligns with the implementation and impact of the *CTOP Act* 92 of 1996, which expanded legal access to CTOP services in South Africa. A comprehensive literature search was conducted across multiple online databases, including ScienceDirect, EBSCOhost, PubMed, Sabinet, African Index Medicus and Google Scholar. Additionally, with the assistance of an institutional librarian, relevant articles were identified by using key search terms and Boolean operators were applied to refine and expand the search. To ensure a thorough review, backward and forward reference searching was also employed to identify additional relevant literature. Only studies published in English were included in this review. A comprehensive search strategy was applied, particularly in PubMed, using the following Boolean search string: (‘professional nurses’ OR ‘registered nurses’ OR ‘nurse practitioners’ OR ‘nursing’) AND (‘safe abortion’ OR ‘termination of pregnancy’ OR ‘induced abortion’ OR ‘legal abortion’ OR ‘choice’ OR ‘termination’ OR ‘safe termination’ OR ‘pregnancy’) AND (‘knowledge’ OR ‘experience’ OR ‘perception’ OR ‘attitude’ OR ‘opinion’ OR ‘belief’ OR ‘practice’ OR ‘role’ OR ‘challenges’ OR ‘barrier*’) AND (‘South Africa’).

#### Stage 3: Screening and selection procedures of the study

The review team employed EndNote version 29.0 to identify and remove duplicate records, organising the search results systematically in Microsoft Excel™. The eligibility criteria for study selection were established using the Population, Exposure and Outcome (PEO) Model.^21^ Table 1 outlines the specific inclusion and exclusion criteria applied to determine the eligibility for the study for this scoping review. A two-stage selection process was implemented to refine the initial pool of studies. In the first stage, two reviewers (Vutlhari A. Ndlovu and Masenyani O. Mbombi) independently screened the titles and abstracts to assess their relevance to the review. Studies focusing on CTOP in South Africa were selected. Conversely, studies were excluded if they (1) did not align with the review question and (2) collected data from populations or settings outside the scope of the review. A third group of studies, whose abstracts did not provide sufficient information, was retained for further assessment. In the second stage, the full texts of all selected studies, including those with ambiguous abstracts, were thoroughly examined, and additional relevant publications were included as necessary. Discrepancies between the reviewers following the first round were resolved through discussion with the third author (Masenyani O. Mbombi), who was consulted for arbitration. Ultimately, a total of 21 studies were included in the review.

**TABLE 1:** Summary of eligibility criteria.

Criterion	Inclusion criteria	Exclusion criteria
Participants	Professional nurses, registered nurses and midwives.	Allied Health Professionals (Physiotherapists, Occupational Therapists, Speech-Language Therapists, Radiographers, Dietitians, Clinical Psychologists, Pharmacists and Social Workers) and medical doctors.
		Other categories of nurses, such as staff nurses and auxiliary nurses.
Setting	All studies published in South Africa.	Studies published outside of South Africa.
Study design	Primary studies that address at least one of the specified objectives of this review using observational study designs or qualitative approaches.	Studies that examine the assessment and diagnosis of pregnancy were excluded from this review.
Language	Peer-reviewed and grey literature publications in English.	Peer-reviewed publications in languages other than English.
Outcome	Studies reporting on attitudes and practices, roles and challenges or issues of CTOP among professional nurses.	Studies with insufficient or unclear information.

CTOP, choice on termination of pregnancy.

#### Stage 4: Charting of data

The extracted data from the selected studies include important details such as the authors, publication year, purpose of the study, design, tool used to collect the data (e.g. questionnaire, interviewers), highlights of the key findings and quality appraisal criteria. Online Appendix 1 – Table S1 presents a summary of the 21 studies included in the review following a full-text assessment. Notably, the Mixed Methods Appraisal Tool Version 2018 was used to critically appraise all included studies for elements such as clear research questions. Thus, the collected data allow addressing the research questions.^22^ This appraisal tool was selected for its established validity and reliability. However, no studies were excluded based on their quality ratings.

#### Stage 5: Collating, summarising and reporting results

This scoping review aimed to synthesise the available literature on nurses’ involvement in the provision of CTOP services in South Africa, using the Socio-Ecological Framework as a guiding lens. Originally proposed by Bronfenbrenner, this framework offers a multi-level perspective on the various factors influencing nurses’ roles and challenges they face in providing CTOP services.^23^ The findings were synthesised using a narrative-qualitative approach, providing a detailed and comprehensive summary of the factors associated with roles and challenges faced by nurses in providing CTOP services as reported in the included studies.

### Ethical considerations

While ethical clearance is not typically required for reviews that utilise secondary data and do not involve direct engagement with human participants, this article forms part of a master’s dissertation that was granted ethical approval by the University of Limpopo Turfloop Research Ethics Committee (TREC) (Reference: TREC/92/2025: PG).

## Results

**FIGURE 1:**
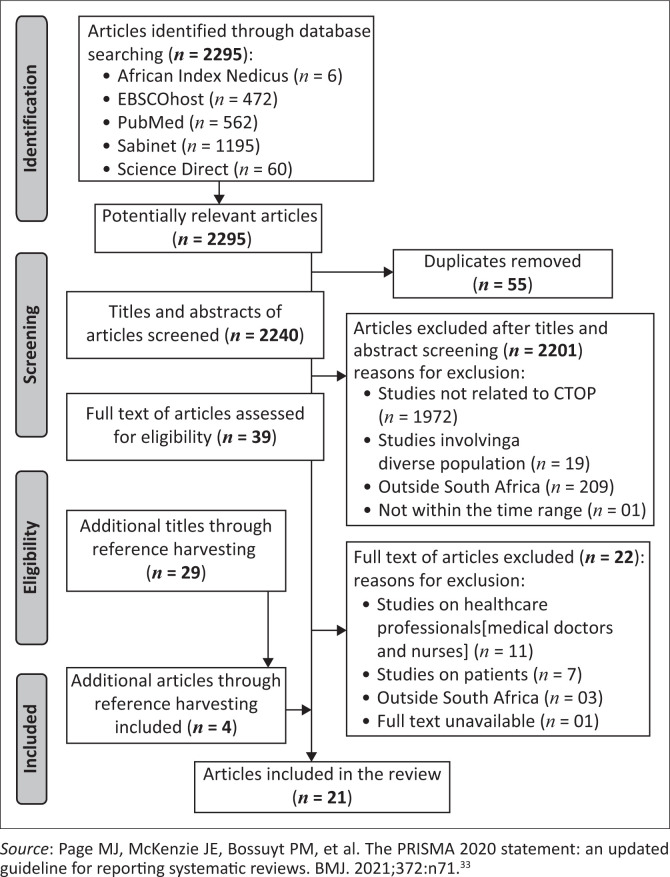
Preferred reporting items for systematic reviews and meta-analyses diagram.

**FIGURE 2:**
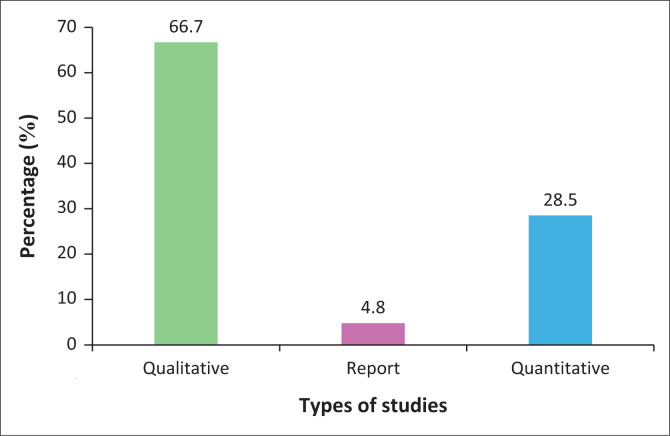
Type of studies included in this review.

This scoping review began with an initial pool of 2295 studies, which was systematically narrowed to 21 that met the inclusion criteria, as shown in Figure 1. In the preliminary phase, titles and abstracts were screened to assess their relevance to the study’s scope, resulting in 39 records selected for full-text evaluation. Of these, 22 were excluded for reasons such as being beyond the study’s scope, focusing on healthcare professionals other than nurses, examining patients receiving CTOP services or being conducted outside South Africa. Additionally, 29 further studies were identified through reference harvesting, including both backward and forward searches. The studies included in this review, published between 1998 and 2023, provided diverse and in-depth insights into the multifaceted experiences of nurses involved in CTOP services, highlighting emotional and moral challenges, interpersonal dynamics, organisational constraints, sociocultural influences and legal gaps. As shown in Figure 2, most of the studies (66.7%) employed qualitative methods, reflecting a strong emphasis on obtaining in-depth, contextually rich insights. Quantitative studies accounted for 28.5%, while a single report-based source represented 4.8% of the total. This distribution underscores the predominance of qualitative approaches in exploring the topic. To the best of the researchers’ knowledge, this is the first review to specifically examine these dimensions of nurses’ roles and challenges in CTOP since the implementation of the *CTOP Act 92* of 1996. Guided by the Socio-Ecological Framework, six themes emerged from the analysis as presented in Table 2.

**TABLE 2:** Summarised findings of the review.

SEF level	Theme	Codes
Individual	Emotional and psychological burden	Emotional distress and trauma
		Moral, religious and ethical dilemmas
		Burnout and professional fatigue
Interpersonal	Stigma and interpersonal strain	Alienation and lack of collegial support
		Labelling and judgemental attitudes
Organisational	Systemic and structural barriers	Inadequate infrastructure, space and equipment
		Poor referral systems and follow-up mechanisms
		Contradictions in resource perceptions
	Training and support deficiencies	Lack of CTOP-specific training
		Limited emotional and managerial support
Community	Community discourses	Community stigma and moral judgement
		Poor public understanding of CTOP
Policy and structural	Shifts in attitude and professionalism	Legalisation versus continued social stigma
		Recognition of reproductive rights and professional evolution

SEF, Socio-Ecological Framework; CTOP, choice on termination of pregnancy.

### Individual factors

The literature identifies that nurses face significant emotional and psychological burdens at a personal level. These include emotional distress and trauma, moral, religious and ethical dilemmas, as well as burnout and professional fatigue. Nurses experience negative emotional, cognitive and behavioural reactions, including emotional distress and frustration.^24,25^ Religion was frequently cited as a central source of tension, when abortion is perceived as a sin or morally wrong.^26^ Many nurses experience guilt, anxiety and religious conflict related to their involvement in CTOP services.^27^ Furthermore, the overwhelming workload and lack of support often lead to burnout and fatigue.^6^ The internal conflicts among nurses impact the provision of the CTOP services in healthcare facilities.

### Interpersonal factors

At an interpersonal level, nurses reported strained relationships and social stigma from peers, colleagues and communities. These interactions are characterised by moral judgement, name-calling, alienation and exclusion from professional and social circles. Nurses describe being labelled as murderers or immoral, which contributes to their emotional isolation and internal conflict.^28,29,30^ Clients themselves are often categorised by nurses as ‘worthy’ or ‘unworthy’ of support, reflecting the moral judgements prevalent within interpersonal dynamics.^31^

### Organisational factors

Numerous studies highlight structural and organisational barriers within healthcare facilities. These include inadequate infrastructure, shortage of trained staff, insufficient equipment and supplies and the lack of dedicated, private spaces for CTOP services.^7,32^ Referral systems are often described as fragmented, and follow-up services are inconsistent.^34^ Despite the legal status of CTOP, many nurses feel unsupported by facility leadership and management.^35^ Additionally, nurses frequently cite a lack of training and professional development on CTOP, further compounding their challenges.^24,36^ Emotional support structures such as debriefing services are available but not widely accessed or effectively utilised.^37^ Nurses also lack of support from managers and doctors.^38^ With regard to the organisational challenges, the study indicates that nurses often face shortages of equipment, lack of support and staff when providing CTOP services.^6^ The management often downplays requests for sufficient resources, equipment, staff and training and development.

### Community factors

Community-level factors reflect the role of sociocultural stigma and public misinformation. Nurses note widespread moral condemnation from the public, often driven by cultural and patriarchal norms.^36,39^ Misunderstandings about the *CTOP Act* and reproductive health rights are common, leading to judgemental attitudes towards both service providers and users.^36^ Limited community education on contraception and legal abortion options results in delays in seeking care, thereby complicating the work of nurses.^30^

### Public policy factors

At the policy level, the review found a disconnect between legislation and implementation. Although CTOP is legally permitted in South Africa, many nurses continue to experience societal stigma and institutional limitations that restrict their ability to provide care effectively.^27^ However, some studies point to emerging shifts in professional identity, where nurses embrace client-centred, rights-based approaches to care, acknowledging CTOP as a public health necessity and legal right.^38,40^ Political leadership was also identified as crucial in creating a supportive environment for reproductive rights.^39^

## Discussion

This article aimed to examine and map the existing evidence regarding the roles and challenges faced by nurses in the CTOP services in South Africa, utilising a socio-ecological framework. The study reveals that the involvement of nurses in CTOP services is shaped by a variety of factors. On a personal level, the review underscores the fact that nurses frequently confront emotional distress and trauma, as well as moral, ethical and religious dilemmas. These findings are consistent with those observed in other regions of Africa. For example, a study conducted in Nigeria found that some healthcare professionals strongly oppose the practice of CTOP, expressing that their role is to heal rather than to take life.^41^ These healthcare professionals contend that providing CTOP services equates to the act of taking a life. The moral obligations and religious dilemmas are consistent across other healthcare professionals involved in the provision of CTOP services. Another study conducted in South Africa indicates that healthcare professionals, on a personal and religious level, view CTOP as murder, the destruction of human life and a sin.^42^ Recent literature in South Africa reports that healthcare professionals have little knowledge of CTOP services and still deem CTOP as an unacceptable practice.^2^ Drawing from trends of studies published in South Africa since the introduction of the *Choice of Termination Pregnancy Act* 92 of 1996, it seems that the duty of TOP was imposed on clinical practice without proper consultation with healthcare providers. This is supported by the notable trends that healthcare professionals felt that their personal and religious beliefs were not considered, as they were expected to provide the CTOP services as part of their scope of practice. This results in their feeling antagonistic towards and dissatisfied with their jobs. Consequently, this compromises the provision of quality CTOP services.^31,42^ However, inconsistencies in CTOP views are noted globally in the North and South. Nicholson, Slade and Fletcher indicate that nurses were unconditionally accepting of the CTOP, but that they felt that CTOP was used as a form of contraception.^43^ This results in their feeling frustrated and having difficulty in supporting women’s decisions. Despite the inconsistencies in the global North and South, similarities were noted among the healthcare professionals. All healthcare professionals were accepting of CTOP and displayed empathy, particularly to the women whose pregnancy resulted from incest or rape or whose termination was necessitated by medical indications.^6,24,41,42,44^ Overall, despite the moral and religious dilemma, the existing literature illuminates how various reasons for CTOP shape the perception and attitude of nurses.

In addition to the internal conflicts faced by nurses with regard to the provision of CTOP services, the review findings are consonant with the Socio-Ecological Framework that the behaviour of nurses towards CTOP is anchored in other problems at multiple levels, not only at the individual level but also at organisation, policy and community levels. Scholars have affirmed that the negative attitudes towards and unacceptable practice of CTOP services are influenced by external factors such as a shortage of resources and equipment and a lack of training and professional development.^2,36^ This is similar to the review findings that reveal that the practices of CTOP services among nurses are negatively impacted by factors at organisational, community and policy levels. Among the factors are lack of support, community and societal stigma and inadequate infrastructure.^27,29,37,39^ The review identifies the profound weight the external factors at community, organisational and policy levels have on shaping the behaviour and attitudes of nurses towards CTOP. Drawing from both the review findings and the Socio-Ecological Framework, the current study underscores that the professional integrity and agency of nurses regarding the acceptability and provision of quality CTOP services in South Africa is embedded within networks of personal beliefs, institutional barriers and broader societal and community influences. The nurses attempt to navigate these dynamics and to maintain respect towards their religious and moral obligations. Despite the identified challenges, these findings advocate for the need to prioritise the provision of reproductive healthcare services such as CTOP. This could be achieved by the provision of adequate resources and training for nurses to optimise the quality of CTOP care. Moreover, nurses and community stakeholders should be included in policy development on CTOP services to mitigate the community stigma towards CTOP and to enhance community and society awareness and understanding of CTOP. As reflected in the findings, CTOP services are sometimes provided in an attempt to save women’s lives. Therefore, the active participation of nurses and community members in psychologically focused awareness campaigns on the CTOP is essential, as it may contribute to reducing mortality associated with backstreet and self-induced abortions in South African communities. Existing research emphasises the influence of community and nurse power dynamics on women’s decisions regarding pregnancy termination.^17^ These dynamics often lead women to choose unsafe methods, thereby exposing themselves to serious risks such as sepsis or even death.

### Strengths and limitations

One of the notable strengths of this review is its application of the Socio-Ecological Framework, which offers a nuanced understanding of the various factors that influence the provision of CTOP services across multiple levels. However, a potential limitation lies in the possibility that some relevant studies may have been overlooked due to variations in the terminology used to describe CTOP services and the roles of nurses. Furthermore, the review focused exclusively on publications in English, which may have excluded valuable research published in other official South African languages, thereby limiting linguistic diversity and potentially missing critical context-specific insights.

## Conclusion

The study elucidates the interplay of individual, interpersonal, community, institutional and policy factors in shaping nurses’ behaviour towards the provision of CTOP services to women in South Africa. Religion, moral obligation, community stigma, insufficient resources and lack of support negatively impact nurses in the provision of quality CTOP services. Efforts to combat community stigma and improve awareness and understanding of CTOP services at the community level should be implemented. This calls for the development of customised, sensitive community-based and workplace initiatives to improve and support nurses in the provision of CTOP services.
